# The Philosophic Spirit in Science1Opening lecture delivered at the University of Buffalo, September 13, 1897.

**Published:** 1897-12

**Authors:** Montgomery A. Crockett

**Affiliations:** Buffalo, N. Y.


### BUFFALO MEDICAL JOURNAL.

VOL. XXXVII. DECEMBER, 1897. No. 5.

### Original Communications.

### THE PHILOSOPHIC SPIRIT IN SCIENCE.[1]

BY MONTGOMERY A. CROCKETT, M. D., Buffalo, N. Y.

WHATEVER one's occupation may be there ever exists the danger of forming too narrow conceptions of life and of overlooking or disdaining those things which seem to lie beyond the limits circumscribing our own activities. It is often claimed that the pursuit of science counteracts this tendency toward narrowness; that the student of biology or medicine develops a broadness and liberality of mind in sharp contrast to the mental attitude displayed by those who busy themselves with other lines of work. By no means would I deny that a liberalising influence flows from the study of natural science, but, alas! only too often its effects are not manifest. The pathologist is much too apt to live within his own microscopic field; too frequently the chemist overlooks what lies beyond the reactions in his test-tubes and the physiologist too often expresses scorn for whatever cannot be demonstrated by his experiments or dissections.

You all know the bicycle scorcher, the man whose range of vision is limited by the patch of ground beside his wheel. For him there are no beauties in the landscape and the success of his excursions is measured by the number of miles recorded on his cyclometer. I fear many a man of science is as limited in his view, so busy registering facts in his particular field that he sees no beauty nor meaning in the landscape beyond. A broad view cannot be obtained without effort; it is often a hard climb to reach the uplands and yet we must ascend some height before we can understand the valley in which we live.

"Unless above himself he can
Erect himself, how poor a thing is man."

1. Opening lecture delivered at the University of Buffalo, September 13, 1897.

During the coming academic year most of you here present will be regaled with facts to the utmost limits of your intellectual digestions and upon this opening night I would not be guilty of inflicting upon you a didactic lecture, therefore I have chosen for my subject The Philosophic Spirit in Science. I wish to invite you onto some height where our view may be broad and from which we may observe some of the lights and shadows which give to the fields of science their impressive beauty. If at times you find the path upward steep and difficult, I beg you to have patience, for I trust the grandeur of the prospect will make you forget the hardships of the ascent.

When the subject of philosophy is mentioned the man of science is prone to display symptoms either of impatience or amusement, thereby illustrating how keenness of insight in one direction may be combined with obtuseness in some other, for philosophy and science are bound together by the closest of family ties. Philosophy represents the effort to explain the meaning of things and I imagine there is no one here present who at times has not philosophised. The facts in your life, your obligations and your experiences, with more or less of the material world must rush over you at times and would overwhelm you did you not think about them and introduce into them some sort of order and meaning. Some kind of a philosophy every one who thinks at all must have ; the question is, what kind ? Shall it be a crude, obsolete, self-contradictory affair ? or will you make use of the ground won by that magnificent army of thinkers in the past and join your thought to theirs ?

At the very dawn of reason, man's religion expressed his attitude toward that world in which he found himself and he soon began to ask the meaning of that universe in which he "lived, moved and had his being." The questions which nature forced upon his mind could find no answers without the data furnished by scientific investigation ; thus philosophy, which was "the child of religion, became the parent of the sciences." The philosopher does not claim to possess any superhuman power by which he can unlock the secrets of the universe ; for him the facts of science are the materials with which he works. You, therefore, can understand why philosophy has been called "the science of the sciences" and the Spencerian definition of philosophy as the last and highest unity of scientific knowledge.

It might be urged at this point that the philosopher is engaged upon a hopeless task, for not only does so much remain to be discovered, but no mind can contain the number of facts already recorded. We must frankly admit the human limitations of the philosopher, but is he in any worse plight than the chemist, for instance ? No chemist would claim that he knows all of his subject or that the time will ever come when he can rest from his labors because nothing more remains for investigation. The term chemist simply defines a worker upon a certain problem whose solution is by no means guaranteed. Happily, the philosopher is engaged upon an endless task, for when the final and complete system of philosophy is reached it will mean that all the data of the universe have been obtained and nothing more is left us but to sink into a condition of everlasting inertia. Science, wonderful as are its advances, raises more problems than it settles. Man's curiosity is so deeply reflective that the more he picks up pebbles from the shore of the great ocean of the unknown, the more pressing and numerous become the questions as to their meaning. What makes the philosopher is the appreciation of the problem which he sets himself and the spirit in which he attacks it. The value of that spirit for every scientist is what I wish to indicate.

A few instances taken from the physiology of sensation afford striking examples of the way in which investigations in a scientific field bring us face to face with profound philosophic questions. Suppose you prick your finger with a pin, what happens ? You talk about a nervous impression being carried upward into the brain, but what is meant by that ? You mean that a molecular vibration has been set up in the nerve and that this vibration is communicated from one cell to another until the cells in the brain also take part in the disturbance. Now, this vibration is what we call nervous energy and like all energy or force is some form of motion. Briefly stated, then, what is conveyed to the brain by your pin-prick is simply a mode of motion.

As soon as this motion reaches the brain it gives rise to a certain particular form of consciousness which we designate as pain. Isolate your sensory nerve or administer an anesthetic and your subject will feel no pain. I think you will readily admit that the reality of pain consists in being experienced by me or by some other sentient being, and that if no one in the universe ever experienced it we would be quite safe in saying that pain did not exist. Suppose now that you roll the pin between your fingers ; again a molecular movement in the nerve is propagated to the brain and you have the consciousness of what you call hardness. Now this hardness, like the pain, you know only as a state of your consciousness and yet you proceed to extradite this sensation into a quality of the pin. From an analysis of the mechanism of the two processes why haven't I as perfect a right to say that the pain, as well as the hardness, is a quality of the pin, or, on the other hand, to say that the hardness, like the pain, has no real existence except in being experienced by a conscious mind ?

Again, take the sensations of color and sound. You know what is meant by light and sound waves. These vibrations are transmitted through the optic and auditory nerves and somewhere within the dark recesses of the skull, this motion becomes translated into terms of consciousness. At any point along the line between the outside object and yourself we find nothing but vibrations and mere vibration by itself is neither color nor sound ; until translated into some form of consciousness these vibrations have no more meaning to you than Egyptian hieroglyphics. On what possible ground are you going to sustain the existence of colors or sounds independent of some perceiving mind ? What we call phenomena are products of a something acting upon a self ; we do not see our eyes in the process of seeing nor among the things seen, and in much the same way we overlook the part played by mind in producing the phenomena which we investigate. It is not my purpose to discuss the solutions of any of the difficulties which we have raised, but, on the contrary, to leave you with a keen appreciation of them, so that you can understand how science suggests them. The study of the physiology of sensation prominently brings forward one thought, that after all our world is what we make it, for our mind acts as the interpreter.

The materialist may tell you that matter thinks, but he never yet has succeeded in getting thought and feeling out of motion except by begging the question which he set out to prove. If you ask him what matter is, he proceeds to describe certain forms of consciousness, such as color, consistency, form and the like, so that instead of starting with matter to show you mind, he really has started with mind to show you matter.

From what has been said you will not be surprised that so many of the great philosophers were scientists. Let me mention a few names at random : Descartes, who is often called the father of modern philosophy, was a physiologist of note ; John Locke, author of the famous essay, Concerning Human Understanding, studied medicine; the great Immanuel Kant was the formulator of the Nebular Hypothesis; Lotze, one of the most famous of recent German philosophers, was a physician and wrote extensively upon physiology and pathology. Huxley's scientific work must not make us forget how much he was interested in philosophy and, finally, Herbert Spencer had the technical training of a civil engineer and a wide knowledge in general science besides. A working acquaintance with some branch of science is indispensable for the proper cultivation of scientific method and judgment, but without the true philosophic spirit even the man so trained becomes but a poor philosopher and a shallow scientist.

There is a department of philosophy known as metaphysics which concerns itself with the discussion of what lies at the basis of all science, and I have heard men of science speak of metaphysics as if that subject represented everything that was absurd and useless. But the scientist makes very heavy demands upon metaphysics and it is rather amusing to hear a man condemn a commodity of which he himself is a large consumer. Causation, matter, motion, law, interaction and the like, are fundamental assumptions of all science; they are the coins used in all scientific transactions; metaphysics enquires as to their value and rejects counterfeits; metaphysics represents a struggle towards correct thinking, which is obtained only through persistent and strenuous effort.

Our senses furnish us with the material upon which the mind works and all we know can be expressed only in terms of ideas. When you use the phrase "I have no idea," you mean you don't know. The greatest conceptions of science are not given in sensation, but are hypotheses formed for the purpose of explaining our states of consciousness. We would have a very small world were we confined to the world of sensation. Did one of you ever see those atoms and molecules which play such important rôles in science? No, these are hypotheses which reason makes, in order to explain our experiences with what we call matter. Not only do the senses not confirm, but, at times, they seem to give evidence directly contrary to some of our fundamental assumptions.

Take the theories in astronomy as instances. Our senses tell us that we and all the stars are at rest and they justify the term sunrise and sunset. How astounding to be told that we are on a rotating ball which is hurled through space at the enormous speed of nineteen miles a second! Our senses limit us to appearances and they have been called man's arch deceivers. The visible heavens we see with our eyes and we at once proceed to go behind the sensible phenomena into the domain of reason, where we construct the hypothesis known as the astronomical heavens, in order to explain our sense experience. Tyndall, after pointing out how we habitually form images of the ultrasensible in order to explain phenomena, says, "In their different powers of ideal extension consists, for the most part, the difference between the great and the mediocre investigator."

It is very evident, then, that metaphysical discussions are of vital importance to the man of science, for they consist in reasoning about his hypotheses and testing them in order to find out whether or not they are contradictory and whether they logically fulfill the demands made upon them. It is a pity that the average scientific man is not subjected to a little metaphysical discipline so that he might appreciate the "lively metaphysical postulates rampant amidst his most positive and matter-of-fact notions" and avoid making some rather sorry exhibitions of himself. Huxley well sums up the matter when he says, "Of all the dangerous mental habits, that which school-boys call cocksureness is probably the most perilous ; and the inestimable value of metaphysical discipline is that it furnishes an effectual counterpoise to this evil proclivity. Whoso has mastered the elements of philosophy knows that the attribute of unquestionable certainty appertains only to a state of consciousness so long as it exists ; all other beliefs are mere probabilities of a higher or lower order. Sound metaphysics is an amulet which renders its possessor proof alike against the poison of superstition and the counterpoison of shallow negation."

From these remarks about metaphysics, I think you can understand why the philosophic spirit is so keenly critical and why it may lead to scepticism. The philosopher takes your pet theories, your tenderest beliefs and subjects them to the deepest scrutiny. The tests which he will bring forward come not from the sense world, but from the domain of reason ; he will wish to know whether the hypothesis reasonably explains the sense impressions. The only objections against the atomic theory to which the physicist would give ear would be those questioning its rationality either in respect to its implications or in its relations to the facts demanding explanation. Doubt is not a thing to be dreaded, but an instrument to be used. There are sceptics who doubt merely for the sake of doubting and for them the philosopher has no use. Doubt in itself is no argument against an hypothesis ; the sceptic must meet the demands of rationality quite as completely as the believer.

Descartes began his philosophic investigations by doubting everything which he could, but he did this in order to reach solid ground underneath ; he doubted because, as he says, "I always had an intense desire to learn how to distinguish truth from falsehood, in order to be clear about my actions and to walk surefootedly in this life." A recent writer (Royce) says "philosophical thought that was never sceptical is sure not to be deep. The soul that has never doubted does not know whether it believes, and, at all events, the thinker who has not dwelt long in doubt has no right to rank high as a reflective person. In fact, a study of history shows that if there is anything that human thought and cultivation have to be deeply thankful for, it is an occasional but truly deep and fearless age of doubt. You may rightly say that doubt has no value in itself. Its value is in what it leads to."

It is well for the man of science to imitate the philosopher in the matter of scepticism. I do not need to dwell upon the fact that every new scientific theory passes through the fiery furnace of doubt. Dr. Whewell says that every great discovery passes through three stages. First, people say "It is absurd," then they say "It is contrary to the Bible," finally they say "We always thought so." How much wrangling do we see among scientists arising from "envy, malice and all uncharitableness." We hate to see our pet theories overthrown and are much too eager to attack a rival's hypothesis. Cultivate, then, in all your scientific work, the honest philosophic doubt which seeks to overcome itself ; be sure that your doubt has its origin in the desire "to learn how to distinguish truth from falsehood."

Both popular and scientific thought agree that the universe is not merely an aggregate of separate things but a system. This view is purely philosophic and is reached only by going behind appearance and entering the domain of reason. The law of gravitation, for instance, is an expression of the interaction between all things. The earth's motion is the resultant of the action of every particle of matter in creation ; astronomers have calculated ten or twelve different motions for the earth, depending chiefly on its varying distances from the sun and its relations to the moon. But, under the gravitation hypothesis, we must think of this planet as undergoing an infinite number of variations of movement. The most distant stars must attract in proportion to their masses and in inverse proportion to the squares of their distances ; if true at all, the law of gravitation applies to the smallest particles of matter, and there cannot be the slightest variation in the most distant nebulæ without every planet and sun adapting themselves to the altered conditions. You cannot throw a baseball into the air without disturbing the milky way. The mind reels in its endeavor to grasp such a tremendous hypothesis.

Suppose we limit our view to that of the human body, we find the same idea of a system. You admit at once that every organ of your body is more or less closely related to every other. You know from your study of pathology how disease at one point sets up disturbance in many distant organs; such changes you readily understand because you see them with your eyes, but you cannot stop at that point. If each individual is a system, a unity, every organ exists only as a part of a whole. Not the minutest imaginable change can take place in any organ without every part of that system altering in order to adapt itself to the changed conditions, and however far improved microscopes and technique can carry us toward the detection of these changes, thought must ever carry us beyond in order to meet the logical demand implied in the idea of a system.

Man himself exists only as a part in a system, and in no other way can we study him. Any man is what he is because he is a son or a brother ; the member of a family, of a church, or of a certain political party. He will vary according as he is an inhabitant of the city, the mountains or the plains of a certain country. And his country is what it is because the land and water are distributed in certain ways, the results of certain geologic changes which carry us back to the friendly cover of the nebular hypothesis where we seek rest from mere weariness of thought. There can be no complete nor correct study of man except in his relations to everything else.

> " 'Tis the sublime of man,
> Our noon-tide majesty, to know ourselves
> Parts and proportions of one wondrous whole."

Thus, when we logically follow out the idea of a system, we see that any one thing in the universe is what it is only because every other thing is what it is. Completely to know any one thing necessitates knowing the entire universe. The field of science must be divided because reality can be conquered only piecemeal, but consider the close dependence of one science upon another.

What can physiology do without chemistry and physics? How inextricable is history interwoven with geography. The great characteristic of the philosophic worker is that, however small his field, he constantly keeps before him the idea of the whole. However faithfully a man may work with his microscope or his reagents, if he works without the philosophic spirit he is in great danger of forming incorrect hypotheses because he never sees his work in its larger aspect. Under such conditions he is at best only a useful artisan; he officiates as a hod-carrier to the true scientific builder.

It is not the field of work which makes a man narrow; it is the spirit with which he enters it. It is fatally easy to allow our minds to shrink to the limits of our own little activities and become blighted with the curse of specialism. Of course we all mean to be broad, but the cultivation of the philosophic spirit which views everything as a part in a system is the only safeguard against narrowness. Whoso works in this spirit is a philosopher, no matter what his field of work may be and from that spirit come the great advances.

You all know how the doctrine of evolution has conquered the scientific world and becomes a working hypothesis of inestimable value. Many people think that this doctrine came from Darwin, but that is a mistake. The idea originated in Greek and Roman philosophy; its great promulgator in this century is Mr. Spencer. Such phrases as "survival of the fittest" and "adaptation to environment" are Spencerian not Darwinian. The argument from the variation of plants and animals under domestication, the argument from geographical distribution and the arguments from embryology had all been used by Mr. Spencer before Darwin's book appeared.

Darwin's contribution was the Theory of Natural Selection. He was a most painstaking collector of facts, but there were numerous other men who possessed facts enough. Darwin was animated by the philosophic spirit about which we have just spoken. The larger view gave him the meaning of this vast array of facts so that not only did he formulate the Theory of natural selection, but he saw the place where that theory fitted into the larger hypothesis of evolution. There is no more brilliant example of the philosophic naturalist than Charles Darwin.

From this rapid glance at some of the relations between philosophy and science you may have received the impression that, after all, philosophy owes more to science than science to philosophy.

What, then, did Prof. Caird mean by calling philosophy the parent of the sciences ? That expression is not a mere figure of speech : it defines the real relationship between the two. By an uncontrollable reflex action man's soul responds to the stimulus of the universe about him, and he is driven into questioning and investigating. The philosophic spirit is the expression of this reaction in every elevated, healthy and enquiring mind. Were it not present, science would be wanting in permanent motive force. The theoretic interest of science lies in the attempt to solve the world riddle, and each department of science furnishes but a few data for the answer. It is not uncommon for some shallow thinker to ask in scornful tones, "What is the use of all your philosophic and scientific theorising ?" And if no material benefit can be demonstrated upon the horizon the philosopher is put down as a dreamer.

About one hundred years ago there came up for discussion the subject of the "spontaneous generation" of life. The subject was not new at that time, but the debate became quite animated and, about the middle of this century, attracted the attention of a prominent French chemist. I presume that many persons of "common sense" deplored the amount of time wasted upon a matter which seemed of so little practical importance and that Pasteur had no conception of what the result would be of his interest in the question. Well, you all know how the science of bacteriology developed from that discussion on infusoria and what the benefits for humanity have been. Sir Joseph Lister (Lord Lister) gives to Pasteur the credit for the only principle which could have conducted him to his antiseptic system. I suppose Benjamin Franklin gave reasons very unsatisfactory to his townspeople why he should amuse himself with kite-flying during a thunderstorm, but the impulse which moved him has resulted in the power-house at Niagara Falls.

If the great scientists had waited to get sight of some material benefits before yielding to the philosophic spirit, our heritage from them would have been small. Men do not take up science as they stake out a mining claim, with the idea of striking it rich. In this school most of you will be called upon to do laboratory work, of whose practical worth no one can assure you in advance, but bear in mind that if the philosophic spirit does not promise you a fortune she does offer you a new world, though you may never appreciate how much that world can produce. Therefore, I say, however much philosophy receives from science, her gifts are but the tokens from a loving child.

Not for one moment should we fail to rejoice in the great and purely practical results which have flowed from scientific research. The extraordinary industrial developments and the lavish favors to humanity at large will be bright flowers in the wreath which science will lay upon the bier of this expiring nineteenth century. But the practical applications of science represent territory already conquered, the real battle line is far beyond on the borders of the unknown and the men who form the Grand Army of Science have their backs to the spoils and their faces to the enemy.

Perhaps in this landscape of scientific fact which we set out to view, its full beauty is not seen with the first hasty glance. Perhaps behind it all lies something which constitutes its chief grandeur. Take this printed page before me. You may subject it to rigid scientific scrutiny, you may analyse the paper, you may study the process of its manufacture, you may work out the mechanism of the typewriter and learn the entire history of printing. All this is very interesting and a perfectly proper field for investigation, but I hope there is something more. I hope there is some idea behind it all, and yet, if so, how do you get the idea ? I part with nothing, I simply make certain signs and those signs become the occasion of your constructing within yourself and from yourself an idea which, I trust, resembles mine. All that we know about anything is the idea which we form of it. What if the features in our landscape have their true reality in the idea behind them and that what we take for the solid objects are, after all, but the shadows ? It would be a ghastly mistake to study the scientific phenomena and miss the idea. On the other hand, is it not a noble conception that whenever scientific experience leads to the underlying ideal truth the scientist has grasped a thought of the Infinite's ?

> "For all experience is an arch where through
> Gleams that untraveled world whose margins fade
> Forever and forever when I move."

Are you afraid that a world of ideas may not be real enough for you ? Ask the missionary to Africa what his real and solid obstacles are ; he will tell you how he has crossed burning sands, braved deadly climates and fought fierce men and beasts, only to be brought to a standstill by an impassable wall of ideas in the minds of a savage people. Look into your own lives and tell me if your heaven and your hell does not lie in your ideas ? Is there no reality in the noble friendship which does not crumble with the passing of the years and which withstands "the slings and arrows of outrageous fortune"? No, the ideal world is but too real, too unyielding. How often would we like to alter it!

But, after all, no one can tell you what you will see when you survey the landscape to which I invited you. The ideas aroused manifest the nature of the gazer; in them lies the difference between the farmer and the poet. The philosophic spirit conducts you to the highest peak and if she does not tell you what to see, she does show you where to look; she warns you not to become sense-bound through scientific work, but to use the mind's eye that you may see the truth which lies behind the senses.

> "The truth which draws
> Through all things upwards; that a two-fold world
> Must go to a perfect Cosmos. Natural things
> And spiritual—who separates these two
> In art, in morals, or the social drift,
> Tears up the bond of nature and brings death,
> Paints futile pictures, writes unreal verse,
> Leads vulgar days, deals ignorantly with men,
> Is wrong, in short, at all points."

452 Franklin Street.

